# Design of a Practical Underwater Sensor Network for Offshore Fish Farm Cages

**DOI:** 10.3390/s20164459

**Published:** 2020-08-10

**Authors:** Graciela Santana Sosa, Judith Santana Abril, Javier Sosa, Juan-Antonio Montiel-Nelson, Tomas Bautista

**Affiliations:** Institute for Applied Microelectronics (IUMA), University of Las Palmas de Gran Canaria (ULPGC), 35015 Las Palmas de Gran Canaria, Spain; gsantana@iuma.ulpgc.es (G.S.S.); jsabril@iuma.ulpgc.es (J.S.A.); montiel@iuma.ulpgc.es (J.-A.M.-N.); bautista@iuma.ulpgc.es (T.B.)

**Keywords:** Underwater Sensor Network (USN), Wireless Sensor Network (WSN), Wireless Power Transfer (WPT), precision aquaculture, offshore fish farm

## Abstract

In this paper, we present the design of a practical underwater sensor network for offshore fish farm cages. An overview of the current structure of an offshore fish farm, applied sensor network solutions, and their weaknesses are given. A mixed wireless–wired approach is proposed to mitigate the problem of wire breakage in underwater wired sensor networks. The approach is based on the serial arrangement of identical sections with wired and wireless interconnections areas. Wireless section alleviates underwater maintenance operations when cages are damaged. The analytical model of the proposed solution is studied in terms of maximum power transfer efficiency and the general formulas of the current in their transmitting antennas and sensor nodes are provided. Subsequently, based on simulations, the effects of parasitic resistance across the network are evaluated. A practical underwater sensor network to reach the 30 m depth with sensor nodes distanced 6 m is used to determine the proposal compliance with the ISO 11784/11785 HDX standard in its normal operation. Taking into account the cable breakage scenario, the results from experiments demonstrate the robustness of the proposed approach to keep running the sensor nodes that are located before the short circuit. Sensor node run time is reduced only 4.07% at most using standard values when a cable breakage occurs at the second deepest section.

## 1. Introduction

Nowadays, the rapid growth of the aquaculture industry requires the use of electronics facilities. In particular, the design and deployment of wireless sensor networks for monitoring and control in fish farms are contributing to reduce operational costs and improve the productivity. In addition, because oceans are hostile environments for any facility, the high maintenance cost is the main drawback for improving productivity. The higher efficiency of the aquaculture industrial production demanded, the more technology introduced. In this sense, fish farming industry is gradually replacing its manual operations by automatic procedures based on electronic devices [1,2,3]. The use of electronics is also motivated to fulfill the government’s health and environmental laws [4,5].

Sensor technology for fish farming has been focused in three main areas, namely, biomass measurements, structural monitoring, and environmental impact evaluation of the industrial activity. For improving productivity, biomass measurements play an important role. The behavior inside the cage of fishes, as a group of individuals of the same species is studied to determine parameters that are related to wellness, such as density population, average physical activity or growth of the specimen, among others. For example, in [6,7], researchers propose tagging a reduced set of individuals, called sentinel fishes, and from studying them infer the evaluation for the complete population. This approach requires at least two receivers strategically placed one at the top of the ocean fish cage and the other at the bottom of the cage.

Sensor networks assist the monitoring of fish farm cage as mechanic structures that are deployed in hostile open sea scenarios [8]. Particularly in Europe, offshore engineering knowledge of fish farms comes mainly from salmon aquaculture. Increasing structural safety design parameters due to the divergence of environmental scenarios [9] is the main target for Atlantic infrastructures. For instance, the structural stress analyses of the critical cases in fjords with frozen nets at cages are different from the Atlantic or Mediterranean ones due to their intricate marine current–velocity profiles. The research presented in [10] is focused on evaluating the hydrodynamic responses of an offshore fish farm to different waves scenarios. They study the forces in the mooring system using several wired arrays of capacitive gauges (load cells). The Internet connection of the cages telemetry monitoring system is the main target of the research presented in [11]. The authors introduce a Low Power Wide Area Network among offshore ocean cages which are distanced up to 2.4 km. In this case, the communications are performed in air at ocean surface level.

The environmental impact of aquaculture activity in marine cages surrounding ecosystems [12] is another research hot topic. In [13], the studies are focused on evaluating the organic matter generated by the fishing farm activity (i.e., nutrients or farm waste among others). For the same application, a strategy to locate the ultrasonic sensor nodes at the seabed taking into account their communications is presented in [14]. The quality monitoring of the water of aquaculture tanks during the feeding process is presented in [15]. The authors propose a low cost underwater wired sensor network to evaluate the feeding process. This approach is based on several wired arrays LDR sensors. All of the studies reveal the need of obtaining a set of measurements distributed throughout the volume of the marine cages and their surroundings. In this sense, the use of sensor network allows for reaching this objective [16,17].

In summary, the massive use of sensors requires the deployment of both a power supply distribution and a data communication network. On the other hand, it is well known that RF-based wireless communications are not capable of reaching the distances required by underwater applications. Nowadays, wireless network solutions are primarily based on ultrasound communications. In addition, the maximum number of sensors is limited by a bandwidth problem. The most popular solution in the literature is to use RF-based wireless networks over the sea surface and the use of wired underwater sensors. From an industrial point of view, the usefulness of such solutions must also be based on the evaluation of deployment and maintenance costs. In other words, their main objective is to reduce the number of working hours of the divers who carry out these tasks.

In this work, we propose a robust underwater sensor network topology in order to mitigate the problem of the wire breakage in wired underwater solutions. The main contributions of this approach are the following:

- A novel mixed wireless–wired topology is introduced and its mathematical model obtained to replace nowadays wired designs and avoid their breakage problems.
- The optimization of the network is performed, maximizing the power transmission, using practical considerations from real life offshore fish farm and describing a practical methodology independent of the number of sections required.
- The performance of the mixed wireless–wired solution has been carried out when considering the occurrence of wire breakage problems demonstrating the robustness of the proposed approach.

The rest of this paper is organized, as follows. In Section 2, an offshore fish farm is introduced. The wireless sensor network design is presented in Section 3. The adopted solution is oriented to improve underwater maintenance. The main aim is to guarantee the energy supply due to the large dimensions of an offshore cage. Therefore, we present the circuital and analytical model for WPT optimization, in Section 4. Circuit optimization, experimental setup and results evaluation are addressed in Section 5, Section 6 and Section 7, respectively. Finally, conclusions are given in Section 8.

## 2. Fish Farm Structure

**Figure 1:**
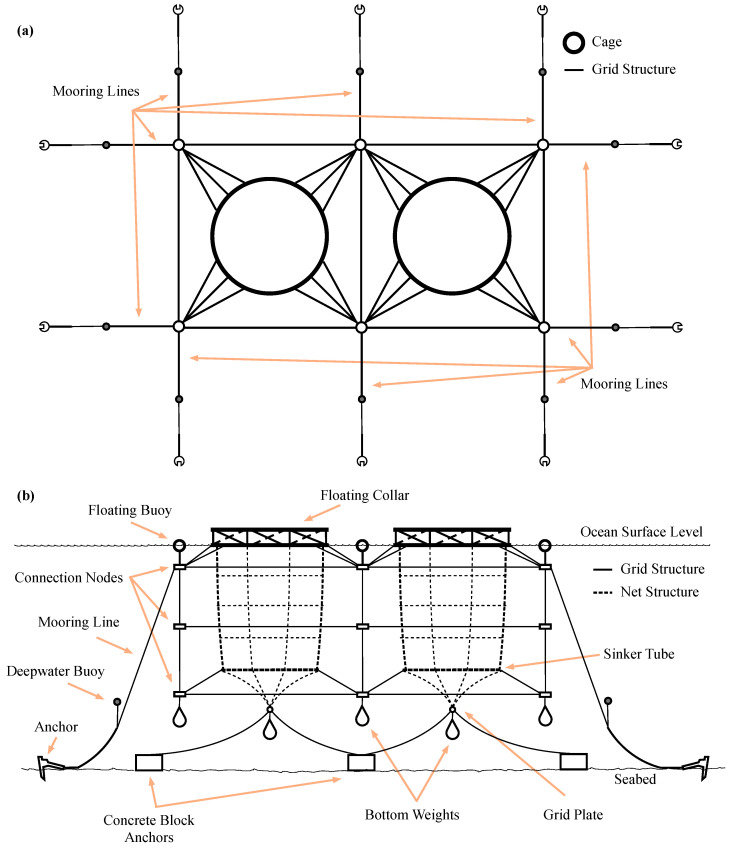
Example of a floating fish farm structure. Top (**a**) and front (**b**) views for a 2 × 1 Grid distribution.

In general, marine fish farm facilities are built grouping several cages following a grid distribution [18]. The grid structure is supported by several mooring elements and buoys. The mooring elements are anchors, shackles, chains, ropes, corner plates, and bridles, among others. Figure 1 shows an example of a floating fish farm.

As is well known, the mission of mooring structure is to keep fixed the location of fish farms, regardless of weather conditions. In this example, the mooring structure is configured as an array of 2 columns in a single line as shown in Figure 1a. Several floating buoys keep the top side of the structure close to the water surface. For underwater fish farms, the floating buoys guarantee the deep placement of the structure. In both cases, they have also the function of warning the presence of the fish farm facilities. The mooring structure is built using a set of ropes and chain lines joined with connection plates or nodes. In addition, the mooring system includes some deepwater buoys to provide structural stability.

The setup that is illustrated in Figure 1a allows the placement of two independent cages. Each cage is placed within a mooring system with its own structure. Basically, each cage is a net with several horizontal and vertical support lines. In this example, Figure 1b shows the cages with dashed lines. At the ocean surface level, the cage net is attached to a floating collar. At the deepest side, there is at least a sinker tube and the net is closed with a grid plate. This bottom grid plate is also connected to several concrete blocks acting as anchors and extra masses as weights.

For safety and environmental considerations of the offshore aquaculture activity, it is desirable to monitor the structural behavior and environmental parameters, along the time. Mostly, preventive maintenance is assisted by structural dynamic analysis, in real live scenarios, by measuring structural movements, accelerations, and applied forces of critical nodes. In particular, monitoring tides and waves damages in real time is desirable. Ecosystem wellness measurement is related to water quality, turbidity, luminance, or nutrient distribution, among others. All of these parameters are obtained by distributed optical sensors. In general, the electronic solution to obtain all of those measurements is based on a network of sensors.

In addition to classical challenges in electronic design, e.g., ultra low power circuits and systems for oceanic floating cages or underwater fish farms, there exist other design key points that increase the solution complexity. In terms of the working scenario, the ocean is, by definition, a hostile environment for electronic devices. The seawater has a high conductivity (0.2 Ω/m) [19]. In addition, RF communications are very limited [20] and finally marine wildlife is aggressive in general [16].

There are other key points to be considered when designing a sensor network for offshore fish cages. The costs of a fish farm (mooring and net system, among others) make its total or partial replacement infeasible. Most of the maintenance costs are related with diving operations because fish cages are partially or fully underwater in the ocean. Due to the high maintenance costs, any new technology to be applied in that framework is evaluated, both in deployment and operation stages. In other words, wireless sensor networks cannot impose new specifications for current fish cages, and its impact in deployment and maintenance costs must be minimized.

## 3. Practical Sensor Network Design

Nowadays, because the underwater RF wireless communications are very limited, most of the research literature is based on acoustic solutions. All of the approaches present some common features. Underwater wireless sensor networks are powered by batteries. The number of available sensor nodes is limited due to the bandwidth of the communication channel. From the cost point of view, the processes of recovering, recharging, and repositioning those sensor nodes increase the production cost.

On the other hand, wired sensor networks have demonstrated their usefulness in land based tanks. This solution overrides the limitation of a maximum number of sensor nodes. However, the wired solutions are not practical in offshore fish cages, fundamentally because of the repairing and maintenance costs. Among others, offshore fish farm cages are damaged due to tides or waves. Their structures have a wide operation range in terms of mobility and deformation. They suffer thefts by other species and endure the natural marine life aggressivety, which, in both cases, create holes in their nets. All of those scenarios lead to cable breakages in the wired approach and the loss of sensor nodes in the wireless solution. Therefore, researchers and industry have been adopted a mixed solution.

**Figure 2:**
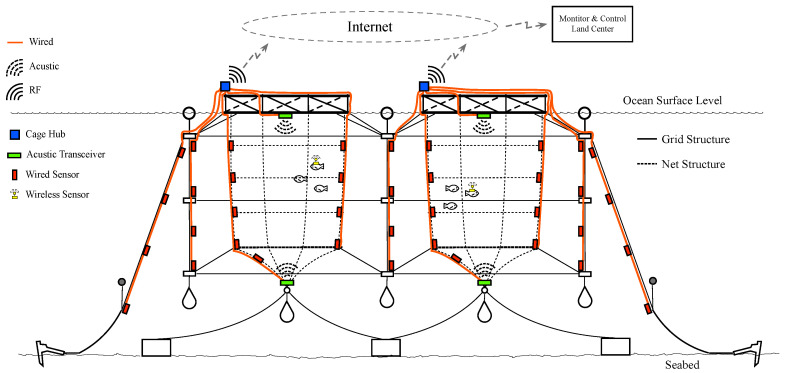
Mixed communication technologies applied to an offshore fish farm sensor network.

Figure 2 presents a mixed communications solution to the previous fish farm example. RF communication is used at the surface level and for underwater ultrasonics or wired communications are proposed. In this approach, at the surface level on each ocean cage, a network hub with RF wireless capabilities is useful. The RF communications transmit all measured data from the wired sensors from the cage to a monitor and control remote center that is located in land. Each hub also includes, in most cases, ultrasonic transceivers to operate with underwater wireless sensors.

In this way, the above mentioned recovery, recharging, and repositioning costs for the wireless sensors are reduced, as the mixed approach minimizes the total number of these type of sensors. The diving operations complexity of deploying the wired sections is reduced using specific cable ties or clamps to fix the sensor nodes and cables to the mooring system or the cage net. However, maintenance or repairing operations over a single sensor requires to replace the whole section. Therefore, it would be desirable to modularize the wired sections in order to reduce these costs. A modular connection system of the sensor nodes would allow maintenance and repair operations to be carried out, exclusively, over the affected areas of the section.

The fastest modular solution is basically to include underwater–electrical plug-in connectors to each sensor node and cable. However, we need to consider that net structure in regular offshore fish farm cages could reach a depth in the range of 35 m to 50 m. Therefore, the connectors must support water pressures from at least 3 atm up to 5 atm without taking into account any further safety consideration. In addition, the size and installation procedures of those kind of connectors increases their manipulation complexity with diving operations and therefore the maintenance cost.

Nowadays, researchers are using the advances in wireless power transfer (WPT) theory applied to battery charging of autonomous underwater vehicle (AUV) [16]. In [21], the researchers propose a 10 m wireless domino power transfer topology that is based on the approach presented in [22]. This could be a possible solution to overcome the excessive usage of underwater connectors. However, the distance between coils is directly proportional to their diameter, as it is presented in both approaches. In addition, the size of receiving coils at the AUV side is close to the size of transmission/domino coil. To reach the 10 m depth, the solution [21] requires seven domino antennas of 3.4 m diameter in the transmission side and a 1.7 m diameter antenna in the receiving side. Those dimensions are not practical in our fish farm cage application.

**Figure 3:**
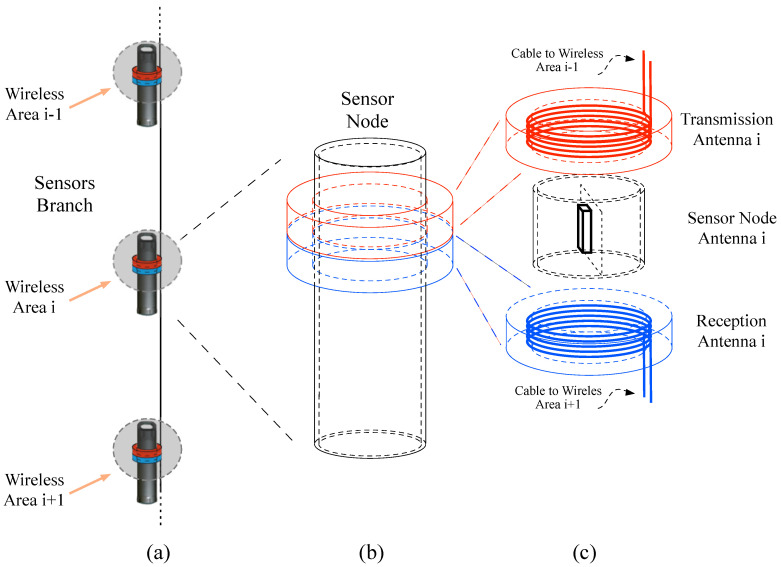
Details of proposed branch for the wireless sensor network; (**a**) wireless areas along network branch, (**b**) sensor node capsule and (**c**) antennas location details.

In this paper, we propose an intermediate solution between domino power transfer and classical WPT theories. Figure 3 shows the details of our proposal. It consists in defining a local wireless area in compliance with the ISO 11784/11785 HDX standard at each sensor node location, as shown in Figure 3a. Each wireless area *i* is connected to the next wireless area i+1 by a cable. In general, each defined wireless area contains at least a transmission antenna and a sensor node. If there exists a wireless area i+1, the wireless area *i* includes a receiving antenna.

The pairs of transmission and receiving antennas fulfill two missions. The first one is to provide energy to the corresponding sensor node, and the second one is to support the energy transport to the next area (see Figure 3b,c for more details). This solution reduces the costs and complexity in comparison with the traditional wired solution from the point of view of the diving maintenance and repairing operations.

This is due to, for example, when, in wired solution, a cable breakage happens, the complete network branch are in short circuit and their nodes disables. Literature and industrial wired solutions require to remove and replace the complete network branch. In similar manner, when a sensor node needs to be replaced the complete network branch must be operated. In our approach, if a cable breakage problem appears, the diving operations are limited to the remove and replacing the affected section or sensor node. Finally, using the ISO 11784/11785 HDX standard as the communication interface at each node allows a diver to verify the sensor node on-site with a portable waterproof reader. This last maintenance operation cannot be performed on wired sensor networks.

## 4. Network Branch Modeling

The proposed approach minimizes the wireless area in order to increase the coupling factor between antennas and the available power along the branch. The size of antenna coils is also taken into consideration in order to keep their operation in short range [22,23,24]. Moreover, the use of a cable allows for increasing the distance between sensor nodes. However, those cables add extra parasitic resistances to the circuit.

**Figure 4:**
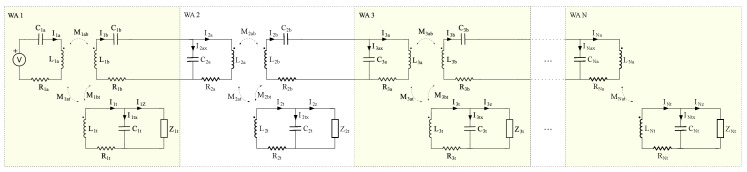
N wireless areas network branch circuital model.

Figure 4 shows the circuital model of the sensor network branch composed of N wireless areas. The first one is labeled as WA 1. In general, all wireless areas incorporate one transmission antenna and two receiving antennas. One of the receiving antennas is used as an energy harvesting device. The other one is used to transmit energy to the next wireless area, as illustrated in Figure 3. In this schema, the first wireless area includes a reader, which is modeled by a voltage generator. Finally, the last wireless area (labeled as WA N in Figure 4) only includes one receiving antenna that corresponds to the sensor node.

In this schematic, all of the components are labeled with a subscript that begins with the number of the wireless area where they are placed. It also includes a letter identifying its behavior, i.e., a, b or t for transmission, receiving or sensor node side, respectively. In the case of mutual inductances, after the wireless area number, the subscript includes letters of modeled transmission–receiving antenna pairs.

For each antenna, its parasitic resistance is modeled and a compensation capacitor is added. Furthermore, each receiving antenna of WA *i* is connected to the transmission antenna of WA i+1 through a cable. Cable parasites are considered within antenna ones for the sake of simplicity.

In the first stage, the reader is modeled by a transmission antenna $L_{1a}$ compensated serially with capacitor $C_{1a}$. In this way, a voltage generator is used as power supply [23]. For each sensor node *i*, its receiving antenna $L_{it}$ is connected in parallel to its compensation capacitor $C_{it}$. This configuration looks like a current source from the point of view of the sensor node load $Z_{it}$.

On the other hand, the receiving antenna $L_{ib}$ is compensated with capacitor $C_{ib}$ in series. The transmission antenna in the next stage observes a voltage supply. In addition, we compensate the transmission antenna $L_{i+1a}$ with $C_{i+1a}$ capacitor in parallel. At this point, we need to take into consideration that the receiving antenna $L_{ib}$ is connected to transmission antenna $L_{i+1a}$ through a bipolar cable. By using this parallel configuration at the antenna $L_{i+1a}$ modeling the cable parasitic capacitance is simplified. In our schematic model, compensation capacitance $C_{i+1a}$ includes the cable parasitic capacitance.

### Analytical Model

Despite all of the sensor nodes being equal, their load impedances are not the same. Note that a sensor node is made up of a battery–powered microcontroller performing diverse tasks on several hardware modules. Therefore, each $Z_{it}$ represents the equivalent impedance of a battery charger connected to a working microcontroller. In other words, each impedance becomes different over time. As a simplification of the behavior model, we assume that each $Z_{it}$ is a capacitor and resistor connected in parallel in order to model the battery and the microcontroller input impedance.

Applying the Kirchoff’s voltage law to the equivalent circuit of Figure 4, we obtained the analytical matrix model of our proposed network branch, which is shown in Equation (1). Note that we present the impedance as a transposed matrix just to increase its readability.

(1) $$\left[\begin{array}{c} V\\ 0\\ 0\\ 0\\ 0\\ 0\\ 0\\ 0\\ \vdots \\ 0\\ 0 \end{array}\right]={\left[\begin{array}{cccccccccccc} \Lambda_{1a} & -j\omega M_{1ab} & -j\omega M_{1at} & 0 & 0 & 0 & 0 & 0 & \;\;\;\;\ldots \;\;\;\; & 0 & 0 & 0\\ j\omega M_{1ab} & \Lambda_{1b} & j\omega M_{1bt} & 0 & \frac{-1}{j\omega C_{2a}} & 0 & 0 & 0 & \ldots & 0 & 0 & 0\\ j\omega M_{1at} & j\omega M_{1bt} & \Lambda_{1t} & \frac{-1}{j\omega C_{1t}} & 0 & 0 & 0 & 0 & \ldots & 0 & 0 & 0\\ 0 & 0 & \frac{-1}{j\omega C_{1t}} & \Lambda_{1z} & 0 & 0 & 0 & 0 & \ldots & 0 & 0 & 0\\ 0 & \frac{-1}{j\omega C_{2a}} & 0 & 0 & \Lambda_{2a} & -j\omega M_{2ab} & -j\omega M_{2at} & 0 & \ldots & 0 & 0 & 0\\ 0 & 0 & 0 & 0 & j\omega M_{2ab} & \Lambda_{2b} & j\omega M_{2bt} & 0 & \ldots & 0 & 0 & 0\\ 0 & 0 & 0 & 0 & j\omega M_{2at} & j\omega M_{2bt} & \Lambda_{2t} & \frac{-1}{j\omega C_{2t}} & \ldots & 0 & 0 & 0\\ 0 & 0 & 0 & 0 & 0 & 0 & \frac{-1}{j\omega C_{2t}} & \Lambda_{2z} & \ldots & 0 & 0 & 0\\ 0 & 0 & 0 & 0 & 0 & \frac{-1}{j\omega C_{3a}} & 0 & 0 & \ldots & 0 & 0 & 0\\ \vdots & \vdots & \vdots & \vdots & \vdots & \vdots & \; & \vdots & \ddots & \vdots & \vdots & \vdots \\ 0 & 0 & 0 & 0 & 0 & 0 & 0 & 0 & \ldots & \frac{-1}{j\omega C_{Na}} & 0 & 0\\ 0 & 0 & 0 & 0 & 0 & 0 & 0 & 0 & \ldots & 0 & 0 & 0\\ 0 & 0 & 0 & 0 & 0 & 0 & 0 & 0 & \ldots & 0 & 0 & 0\\ 0 & 0 & 0 & 0 & 0 & 0 & 0 & 0 & \ldots & \Lambda_{Na} & -j\omega M_{Nat} & 0\\ 0 & 0 & 0 & 0 & 0 & 0 & 0 & 0 & \ldots & j\omega M_{Nat} & \Lambda_{Nt} & \frac{-1}{j\omega C_{Nt}}\\ 0 & 0 & 0 & 0 & 0 & 0 & 0 & 0 & \ldots & 0 & \frac{-1}{j\omega C_{Nt}} & \Lambda_{Nz} \end{array}\right]}^{T}\cdot \left[\begin{array}{c} I_{1a}\\ I_{1b}\\ I_{1t}\\ I_{1z}\\ I_{2a}\\ I_{2b}\\ I_{2t}\\ I_{2z}\\ I_{3a}\\ \vdots \\ I_{(N-1)b}\\ I_{(N-1)t}\\ I_{(N-1)z}\\ I_{Na}\\ I_{Nt}\\ I_{Nz} \end{array}\right]$$

where:

(2) $$\Lambda_{iy}=R_{iy}+j\left(\omega L_{iy}-\frac{1}{\omega C_{iy}}\right)$$

with i∈Z[1,N] and y={a,t}, also:

(3) $$\Lambda_{ib}=R_{ib}+j\left(\omega L_{ib}-\frac{1}{\omega C_{ib}}-\frac{1}{\omega C_{(i+1)a}}\right)$$

with i∈Z[1,N−1]. In addition,

(4) $$\Lambda_{iz}=\frac{1}{j\omega C_{it}}+Z_{it}$$

with i∈Z[1,N].

Finally, we assume that, in Equation (1), all of the currents of the parallel compensation capacitors are computed from others in the circuit. Those components are subscripted with an ending *x* letter in Figure 4. Subsequently, the compensation capacitor currents for the transmission antenna and the sensor node are, respectively:

(5) $$I_{ix}=I_{(i-1)b}-I_{ia}\;,\;\;\;\;\forall \in Z\left[2,N\right]$$

(6) $$I_{itx}=I_{(i)t}-I_{iz}\;,\;\;\;\;\forall \in Z\left[1,N\right]$$

## 5. Sensor Network Optimization

Our interest is focused on optimally supplying energy to all of the sensor nodes. However, it is well known that, in this kind of circuits, there is less energy available, as more wireless areas are between the reader and sensor node [22]. Therefore, the last sensor node is more critical than all previous ones. For this reason, we optimize the transmission power from the point of view of the last sensor node.

Based on Equation (1), we are able to obtain all currents in our proposed circuit. We assume that all parasitic resistances are negligible in order to reduce the mathematical complexity of the last sensor node current formulation. In spite of this discard, we should note that all currents in every sensor node $I_{iz}$ depend on the reflected impedances of the other sensor nodes. Furthermore, as the wireless network branch grows in terms of the number of wireless areas, the analytical solution for the current of each sensor node increases considerably in complexity.

In terms of optimization procedure, there exists an upper bound of the power efficiency in the last sensor node that we can obtain. By definition, this upper bound incorporates less mathematical complexity than the expressions of the sensor nodes currents. This upper bound appears with the best scenario for the last sensor node, which is, a network branch where there are no other sensor nodes. This setup can be derived, in our matrix model presented in Equation (1), assigning zero to the right mutual inductances. In other words, we determine the power efficiency at sensor node N when:

(7) $$M_{iat}=M_{ibt}=0,\;\;\forall i\in Z\left[1,N-1\right]$$

For this upper bound scenario, we obtained the values for each compensation capacitor reducing to zero the reactive energy required at the reader in wireless area 1 from the point of view of the last sensor node ($Z_{Nt}$). All of the transmission antennas $L_{ia}$, from wireless areas 1 to N−1, are compensated as a traditionally serial to serial topology [25], as follows:

(8) $$C_{ia}=\frac{1}{\omega^{2}L_{ia}},\;\;\;\;\forall i\in Z\left[1,N\;-\;1\right]$$

The last transmission antenna $L_{Na}$ at wireless area *N* is only connected to its sensor node antenna $Z_{Nt}$. At first glance, this transmission antenna and its sensor node antenna follow a parallel to parallel configuration. However, we found that the required compensation is:

(9) $$C_{Na}=\frac{1}{\omega^{2}\left(L_{Na}+M_{Nat}^{2}/L_{Nt}\right)}$$

On the other hand, the first receiving antenna is compensated in a traditional way as a serial to serial configuration, which is:

(10) $$C_{1b}=\frac{1}{\omega^{2}L_{1b}}$$

We compensate the other receiving antennas following a serial to parallel configuration without taking into consideration the intermediate sensor node antennas, as follows:

(11) $$C_{ib}=\frac{1}{\omega^{2}\left(L_{ia}+M_{iab}^{2}/L_{ib}\right)},\;\;\;\;\forall i\in Z\left[2,N\;-\;1\right]$$

Similarly, the compensation capacitor $C_{Nt}$ for the last sensor node antenna is determined while using equation:

(12) $$C_{Nt}=\frac{1}{\omega^{2}\left(L_{Nt}+M_{Nat}^{2}/L_{Na}\right)}$$

After setting those resonation conditions, we can determine the currents in the circuit proposed. As mentioned in previous paragraphs, we are interested in maximizing the energy transmission from the point of view of the last sensor node. Equation (13) shows the result of applying those conditions to Equation (1).

(13) $$\begin{array}{c} \left[\begin{array}{c} V\\ 0\\ 0\\ 0\\ 0\\ 0\\ 0\\ \vdots \\ 0\\ 0 \end{array}\right]={\left[\begin{array}{ccccccccc} 0 & -j\omega M_{1a} & 0 & 0 & 0 & 0 & \ldots & 0 & 0\\ j\omega M_{1ab} & -j\omega L_{2a} & j\omega L_{2a} & 0 & 0 & 0 & \ldots & 0 & 0\\ 0 & j\omega L_{2a} & 0 & -j\omega M_{2ab} & 0 & 0 & \ldots & 0 & 0\\ 0 & 0 & j\omega M_{2ab} & \Lambda_{2b}^{\prime } & j\omega L_{3a} & 0 & \ldots & 0 & 0\\ 0 & 0 & 0 & j\omega L_{3a} & 0 & -j\omega M_{3ab} & \ldots & 0 & 0\\ \vdots & \vdots & \vdots & \vdots & \vdots & \vdots & \ddots & \vdots & \vdots \\ 0 & 0 & 0 & 0 & 0 & 0 & \ldots & -j\omega M_{Nat} & 0\\ 0 & 0 & 0 & 0 & 0 & 0 & \ldots & -j\omega \frac{M_{Nat}^{2}}{L_{Na}} & \Lambda_{Nx}^{\prime }\\ 0 & 0 & 0 & 0 & 0 & 0 & \ldots & \Lambda_{Nx}^{\prime } & \Lambda_{Nz}^{\prime } \end{array}\right]}^{T}\cdot \left[\begin{array}{c} I_{1a}\\ I_{1b}\\ I_{2a}\\ I_{2b}\\ I_{3a}\\ \vdots \\ I_{Na}\\ I_{Nt}\\ I_{Nz} \end{array}\right] \end{array}$$

where:

(14) $$\Lambda_{ia}^{\prime }=-j\omega \left(L_{ia}-L_{ib}+L_{(i+1)a}+\frac{M_{iab}^{2}}{L_{ib}}\right),\;\;\;\;\forall i\in Z\left[1,N\right]$$

(15) $$\Lambda_{x}^{\prime }=j\omega \left(L_{Nt}+\frac{M_{Nat}^{2}}{L_{Na}}\right)$$

(16) $$\Lambda_{Nz}^{\prime }=-j\omega \left(L_{Nt}+\frac{M_{Nat}^{2}}{L_{Na}}\right)+Z_{Nt}$$

As an example, for a given network branch with three wireless areas and based on matrix Equation (13), once the compensation values are determined, the current at reader side is:

(17) $${\hat{I}}_{1a}=-{\left[\frac{L_{2a}\left(L_{3a}L_{3t}+M_{3at}^{2}\right)}{M_{1ab}M_{2ab}M_{3at}}\right]}^{2}\frac{V}{Z_{3t}}$$

In a similar way, the current at the sensor node is:

(18) $${\hat{I}}_{3z}=-\frac{L_{2a}\left(L_{3a}L_{3t}+M_{3at}^{2}\right)}{M_{1ab}M_{2ab}M_{3at}}\frac{V}{Z_{3t}}$$

The magnetic coupling coefficient is defined as:

(19) $$M_{iax}^{2}=K_{iax}^{2}L_{ia}L_{ix},$$

with i∈[1,3] and x∈{b,t}. Equations (17) and (18) are rewritten as Equations (20) and (21), respectively:

(20) $${\hat{I}}_{1a}=-{\left(\frac{1+K_{3at}^{2}}{K_{1ab}K_{2ab}K_{3at}}\right)}^{2}\frac{L_{2a}L_{3a}L_{3t}}{L_{1a}L_{1b}L_{2b}}\frac{V}{Z_{3t}}$$

(21) $${\hat{I}}_{3z}=-\frac{1+K_{3at}^{2}}{{\left(K_{1ab}K_{2ab}K_{3at}\right)}^{2}}{\left(\frac{L_{2a}L_{3a}L_{3t}}{L_{1a}L_{1b}L_{2b}}\right)}^{\frac{1}{2}}\frac{V}{Z_{3t}}$$

We determined the last load and power source currents for several network branches with different total numbers of wireless areas using matrix Equation (13) in order to obtain a general formulation of these equations. As a result, we found the following equations for those currents:

(22) $${\hat{I}}_{1a}={\left(-1\right)}^{N}{\left(\frac{1+K_{Nat}^{2}}{K_{Nat}\prod_{j=1}^{N-1}K_{jab}}\right)}^{2}\frac{L_{Nt}\prod_{j=2}^{N}L_{ja}}{L_{1a}\prod_{j=1}^{N-1}L_{jb}}\frac{V}{Z_{Nt}}$$

(23) $${\hat{I}}_{Nz}={\left(-1\right)}^{N}\frac{1+K_{Nat}^{2}}{K_{Nat}^{2}\prod_{j=1}^{N-1}K_{jab}^{2}}{\left(\frac{L_{Nt}\prod_{j=2}^{N}L_{ja}}{L_{1a}\prod_{j=1}^{N-1}L_{jb}}\right)}^{\frac{1}{2}}\frac{V}{Z_{Nt}}$$

Finally, the power efficiency of the upper bound is defined as:

(24) $${\hat{\eta }}_{Nz}=\frac{{\hat{I}}_{Nz}^{2}Z_{Nt}}{{\hat{I}}_{1a}V}=1$$

This last equation demonstrates the perfect adaptation of the entire network. In other words, the proposed formulas for all compensation capacitors exhibit their usefulness.

## 6. Experimental Setup

The theoretical model following Equation (24) was obtained assuming that parasitic resistances are negligible. Now, we will evaluate their influence on the current load. Once the network is adapted, we are interested in keeping those parasitic resistances as low as possible. The effect is a reduction of the available energy at each load.

The functionality implemented inside of the sensor node can be optimized, however our goal in this paper is focused on the optimization of the wireless–wired network. In our case, our sensor node is composed of a microcontroller from NXP and several sensors devices, like an accelerometer, gyroscope, and compass, in order to measure structural behaviors and an optical interface obtain environmental variables like turbidity or luminance among others. After building our sensor node, we obtained an average power consumption in several corner cases and chose the worst case of power consumption.

**Figure 5:**
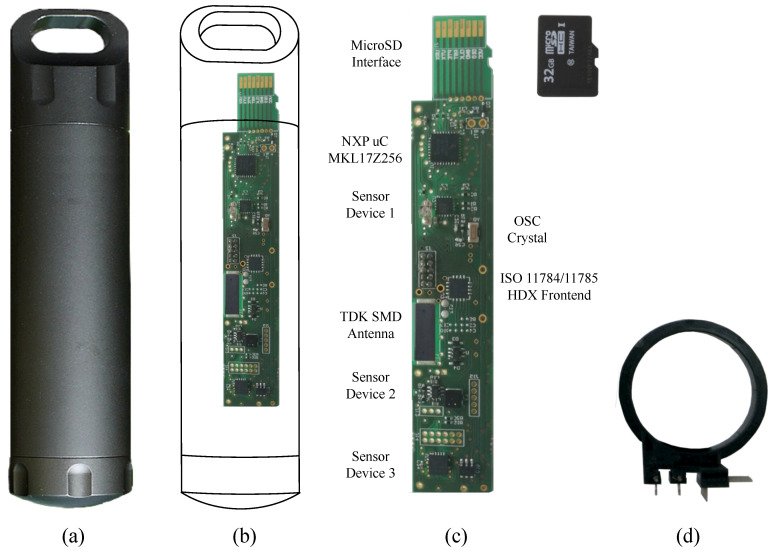
Sensor node used in the experiments: (**a**) Sensor node capsule, (**b**) mainboard and sensor node size comparison, (**c**) Sensor node mainboard details, and (**d**) Top view of the transmission and receiving antenna PLA plastic holder.

For this reason, we used a sensor node made with commercial off-the-shelf (COTS) devices. Figure 5 shows the sensor node used to evaluate our proposal. It uses a military grade aerospace heavy duty aluminum capsule (See Figure 5a). Inside of the capsule, all electronic parts have been placed and its holder has been printed using Polyactic Acid (PLA) plastic. Figure 5b compares the sensor node mainboard and the capsule sizes. The mainboard photo is shown in Figure 5c. This figure also includes a microSD card as size reference. Finally, Figure 5d shows the top view of the transmission and receiving antenna PLA plastic holder used in our experiments.

In our proposal, we use the same antenna model for transmission ($L_{ia}$) and reception ($L_{ib}$) due to us being interested in a modular system. We choose a circular inductor of 452 μH as antennas to operate at 134.2 kHz following the ISO 11784/11785 HDX standard (see Figure 5d). In addition, we used a twisted pair cable to connect the receiving antenna ($L_{ia}$) with the transmission antenna ($L_{i+1a}$). Its conductors are made with 23 AWG solid bare copper wires. Its mutual capacitance is 50.52 pF/m and its loop resistance is 124 Ω/km.

For the sensor node antenna, the HDX tranceiver used is the TMS37157. Additionally, we selected a SMD ferrite core inductor of 2.66 mH. This antenna is labeled as TDK SMD Antenna in the sensor node mainboard, see Figure 5c for details. We measured that, in the worst case, the maximum consumption of the sensor node is equivalent to a load of 15 kΩ.

## 7. Results Evaluation

### 7.1. Adaptation and Parasitic Resistances

Obtaining an analytical formula of how each parasitic resistance reduces the total available power follows. Assuming that $R_{1a}\neq 0$ in Equation (1), then the load current for last sensor ($I_{3z}$) in our example with three stages is:

(25) $${\hat{I}}_{3z}\left(R_{1a}\right)=\frac{L_{2a}\left(L_{3a}L_{3t}+M_{3at}^{2}\right)M_{1ab}M_{2ab}M_{3at}V}{L_{2a}^{2}{\left(L_{3a}L_{3t}+M_{3at}^{2}\right)}^{2}R_{1a}-M_{1ab}^{2}M_{2ab}^{2}M_{3at}^{2}Z_{3t}}$$

The greater the number of stages, the more complex the expression for the last sensor load current $I_{3z}$. In this example for three stages, the last load current for $R_{2a}\neq 0$ is:

(26) $${\hat{I}}_{3z}\left(R_{2a}\right)=\frac{L_{2a}\left(L_{3a}L_{3t}+M_{3at}^{2}\right)M_{2ab}M_{3at}V}{M_{1ab}L_{2a}{\left(L_{3a}L_{3t}+M_{3at}^{2}\right)}^{2}R_{2a}+M_{1ab}M_{2ab}^{2}M_{3at}^{2}\left(jR_{2a}/\left(\omega L_{2a}\right)+1\right)Z_{3t}}$$

As we expected, parasitic resistances $R_{ia}$ reduces the current at the last sensor node and, therefore, the available energy. In our case, the current decrement follows a 1/x law. The current at the last sensor node depends on the values of mutual inductances, transmission, receiving, and sensor node antennas.

We calculated the mutual inductances for the proposed setup while using FEM. The computed value for a complete alignment of antennas, with transmission and reception, when they are placed within a distance of 200 μm is $M_{iab}$ = 316.16 μH. That inductance is providing an equivalent coupling factor of $K_{iab}$ = 0.6996. The mutual inductance between the transmission and sensor node antennas is $M_{iat}$ = 73.5 μH and the equivalent coupling factor is $K_{i}$ = 0.06632.

In those conditions, the $R_{2a}$ term in the denominator of Equation (26) is greater than the modulus of $Z_{3t}$ in four orders of magnitude. From the point of view of parasitic resistance, its influence is greater in the first stages than in the last. The practical values of parasitic resistances for the proposed antennas are lower than 9.3 Ω. On the other hand, the equivalent impedance for the sensor node load is lower than 15 kΩ, as was indicated in Section 6. For those impedances, the $R_{1a}$ term in the denominator of Equation (25) is 27.9 times greater than the $Z_{3t}$ term.

We simulated our proposed circuit with the parameters shown in Table 1 in order to evaluate the influence of parasitic resistances. In this setup, there are five sensors (*N* = 5) placed every six meters from each other. This means that our proposal requires a 6 m cable section between every receiving and transmission antenna and the total length of the network branch is 30 m.

Based on our proposed Equations (8), (9), (11), and (12), we obtained all compensation capacitances. The computed values for $C_{ia}$ and $C_{ib}$ are similar (close to 3.1 nF) and the compensation capacitor for the sensor node antenna ($C_{it}$) is 526.489 pF. In this experiment setup, the sensor node load $Z_{it}$ is modeled with a resistor of 15 kΩ.

For this setup, the selected 23 AWG cable produces a parasitic loop resistance of 0.744 Ω. The worst case is to assume that this cable resistance is computed within the transmission antenna resistance ($R_{ia}$). In that case, the previous considerations are still valid. The parasitic resistance of the cable is a key optimization parameter for maximizing the power transfer to the load of the last network branch. The higher the wire gauge, the more power can be transferred. For example, using a 8 AWG cable, the parasitic loop resistance is around 0.024Ω.

Finally, in this setup, we can assume that the parasitic resistance of the transmission antenna is mainly determined by its wire strand class. In this sense, when this antenna is built with a 38 AWG its parasitic value $R_{ia}$ is 9.3 Ω. On the other hand, when a 23 AWG is used, this value decreases to 248.16 mΩ.

**Figure 6:**
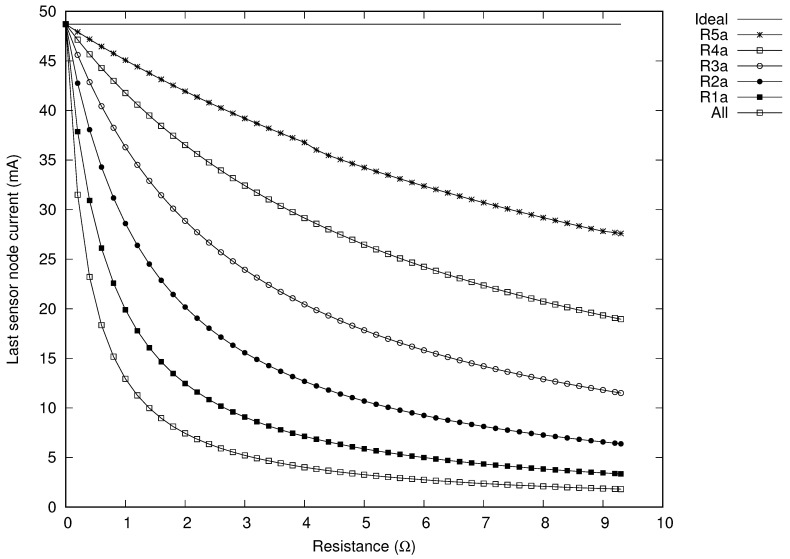
Simulation results of the last sensor node current dependency for a five stages network branch.

Figure 6 presents the results obtained when only one of those parasitic resistances is taken into consideration. Additionally, it included the behavior of the last sensor node when all transmission parasitic resistances are evaluated.

The simulation results that are shown in Figure 6 depict, in the horizontal axis, the variation of the parasitic resistance between 100 mΩ and 9.3 Ω when the reader transmits a sinusoidal signal of 5 Vpp at 134.2 kHz. The vertical axis presents the last sensor node current measured in mA. The curve at the top in this Figure 6 shows the circuital behavior when no parasitic resistances are considered. This scenario defines the current upper bound. We obtained, for the proposed setup, that the maximum current is 48.71 mA. The curve at the bottom represents the worst case, which is, when all parasitic resistances from all the transmission antennas are taken into consideration. There is a current of 1.864 mA when all parasitic resistances are equal to 9.3 Ω. The other curves represent the current obtained when a single parasitic resistance from each transmission antenna is considered.

In the worst case scenario, a fully charged battery of 3 mAh allows 1000 mins of sensor node continuous operation—a microcontroller working at ultra low power run mode with all peripherals turned on. In our setup, the optimized current charges this battery in less than 97 minutes for the last sensor node (*N* = *i* =5). This charging time is only 10% of the total worst case execution time.

The influence of the parasitic resistance of the transmission antenna is greater when it is closer to the network branch reader (lower stages), as observed in Figure 6. On the other hand, the proposed topology for the network branches made the current for the last sensor node insensitive for all parasitic resistances of the receiving antennas ($R_{ib}$). However, the parasitic resistance of the sensor node antenna introduces an attenuation in its current. Following our example for a three branch network, Equation (27) presents the value of the sensor node current when its antenna parasitic resistance ($R_{3t}$) is taken into consideration.

(27) $${\hat{I}}_{3z}\left(R_{3t}\right)=\frac{L_{2a}\left(L_{3a}L_{3t}+M_{3at}^{2}\right)}{M_{1ab}M_{2ab}M_{3at}}\frac{V}{\left(L_{3a}L_{3t}+M_{3at}^{2}\right)R_{3t}+\left(jL_{3a}R_{3t}/\omega +\left(L_{3a}L_{3t}+M_{3at}^{2}\right)\right)Z_{3t}}$$

The SMD antenna used in the sensor node has a parasitic resistance of 26 Ω. Using the designed values in our proposal, the imaginary term $L_{3a}R_{3t}/\omega$ is 88.7 times lower than the term $L_{3a}L_{3t}+M_{3at}^{2}$. On the other hand, the load impedance is greater than the sensor node antenna parasitic resistance by approximately three orders of magnitude. Therefore, the influence of this parasitic resistance is neglected, as it was assumed.

### 7.2. Frequency Response

The next step is to determine the frequency response of the proposed network branch. We define a new experiment using the parameter values presented in Table 2. In this new experiment setup, we include all of the sensor nodes working at the same time. Additionally, moreover, all the sensor nodes have the same parameter values. In addition, we assume that cables, transmission, and receiving antennas use the 23 AWG standard. In this scenario, the transmission and receiving parasitic resistances $R_{ia}$ and $R_{ib}$ are 620.16 mΩ, which include the half loop resistance of their connected wires.

**Figure 7:**
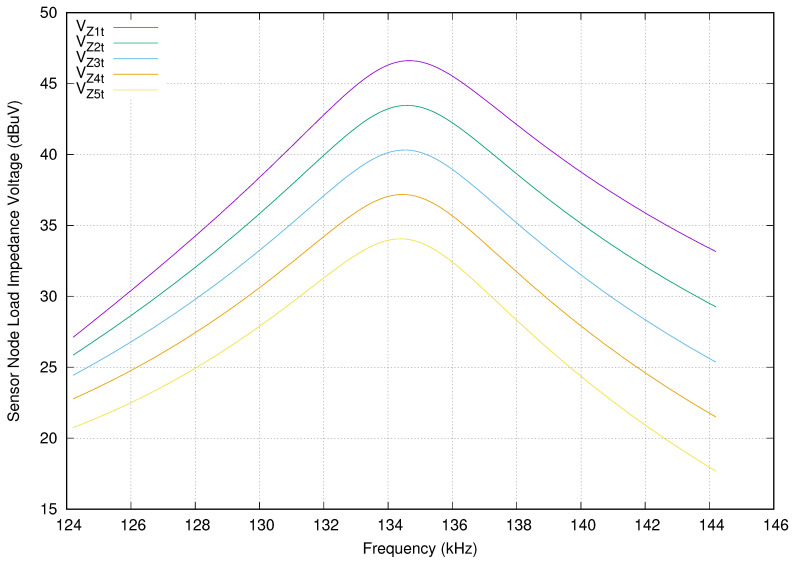
Sensor node load voltage in terms of frequency response when all sensor nodes are connected.

Figure 7 shows the voltage simulated in each sensor node load in terms of the frequency. The vertical axis presents the voltage in dBuV units and the horizontal axis shows the frequency in kHz. As expected, the received power is maximum at 134.2 kHz for all sensor nodes. In this case, the maximum voltage obtained is 46.46 dbuV for the first sensor node and for the second one is 43.35 dBuV, which is −3.11 dB. The deepest sensor node receives 34.03 dBuV. In comparison with the first sensor node, it is 12.43 dB smaller. Each wireless stage reduces the total received voltage −3.11 dB on average. In other words, every sensor node receives half the power than the previous one.

**Figure 8:**
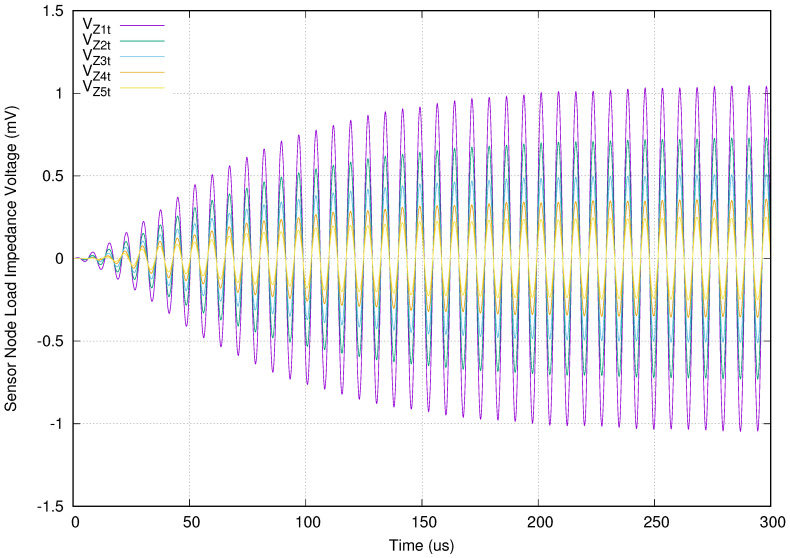
Transient behavior when all sensor nodes are connected.

Once checked the frequency response of the proposed solution, it is mandatory to verify the transient behavior using the same setup shown in Table 2. Figure 8 presents its transient simulation. The vertical axis presents the voltage in mV at the sensor node load ($Z_{it}$). The load in this experiment setup is a 15 kΩ resistor. Additionally, the horizontal axis depicts the simulated time, measured in microseconds.

In this Figure 8, we can see that all of the sensor nodes voltages have the same phase after 50 μs. This confirms the correct choice of compensation capacity values. Other important key is to determine how long the transient behavior is. In this experimental setup, the received voltages are stable after 250 μs. This represents 33.55 cycles of the resonance frequency. The TMS37157 transceiver used in our sensor node requires minimum time, to charge in a batteryless operation, of at least 20 ms of stable received signal. This measured time only represents 1.25%.

### 7.3. Normal Operation

**Figure 9:**
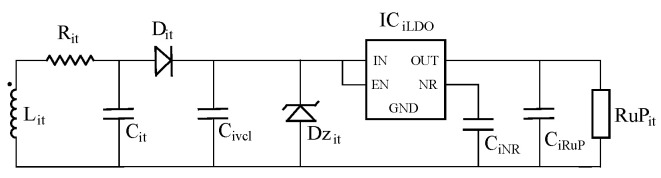
Batteryless sensor node equivalent circuit.

Now, we assume that all sensor nodes are working in batteryless mode. In this case, Figure 9 shows the equivalent circuit model of the batteryless sensor node. It takes into account the power management circuitry of the TMS37157. In this model, the sensor node antenna $L_{it}$ is connected in parallel to its compensation capacitor $C_{it}$. They drive a charge capacitor $C_{ivcl}$ using diode $D_{it}$. The maximum voltage of this charge capacitor to 6V is limited using the Zener diode $Dz_{it}$. Subsequently, a low dropout regulator (LDO, labeled as $IC_{iLDO}$) to supply a nominal voltage of 2.7 V is included. In addition, capacitors $C_{iNR}$ and $C_{iRuP}$ are required to stabilize LDO outputs. Finally, resistor $RuP_{it}$ models the microcontroller power consumption. Table 3 presents the values of the components of the sensor node model.

This experimental setup uses the compensation capacitors, transmission, and receiving antennas from Table 2. A sensor node is available in each wireless area of a five-stage network branch (*N* = 5). Where, all sensor nodes are working in batteryless mode. In this setup, the reader generates a sinusoidal signal at 134.2 kHz for a period *T*. The energy stored in $C_{icvcl}$ allows to work extra time after finishing the energy transmission time period *T*. In this experiment, we set this value to 4 ms.

**Figure 10:**
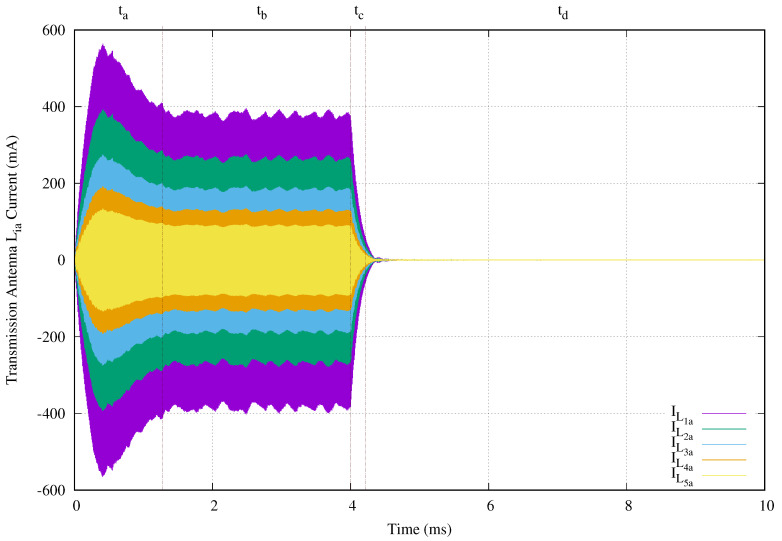
Currents in each transmission antenna of a network branch with five stages when a 4 ms tone of 134.2 kHz is applied.

Figure 10 shows the transmission antenna currents $I_{L_{ia}},i\in \left[1,5\right]$ for this experimental setup. The vertical axis gives the antenna current in milliamperes and the horizontal axis presents the time in microseconds. This behavior can be divided in four time periods, labeled as $t_{a}$, $t_{b}$, $t_{c}$, and $t_{d}$, at the top of the image. During the two periods, $t_{a}$ and $t_{b}$, the power transmission signal is present ($t_{a}+t_{b}=T$). And periods $t_{c}$ and $t_{d}$ correspond to the sensor node behavior when the power transmission signal is absent.

In period $t_{a}$, the transmitted energy is mainly used charging capacitor $C_{ivcl}$. The current behavior at the second period is due to the LDO regulation and the microprocessor power consumption. Once the energy transmission finishes at 4 ms, the stored energy in compensation capacitors, transmission, and receiving antennas begins to discharge through sensor nodes and all parasitic resistances. This behavior comprises the $t_{c}$ period. Finally, $t_{d}$ correspond to the discharge of the accumulated energy in capacitor $C_{ivcl}$.

**Figure 11:**
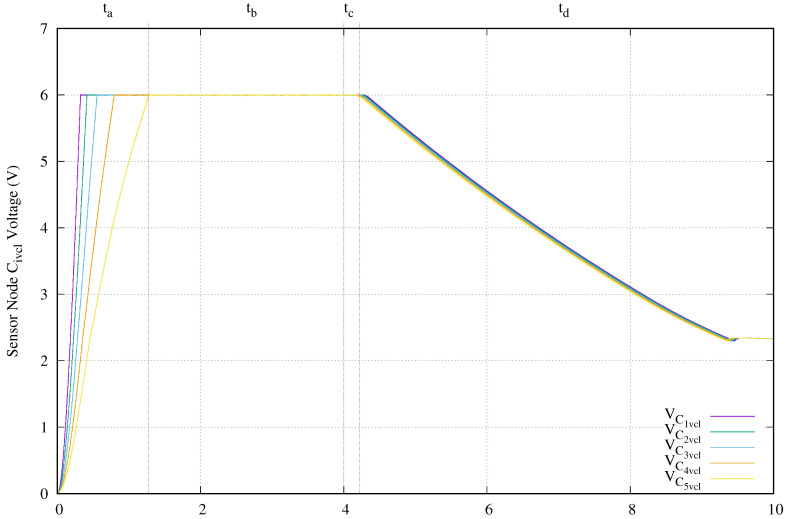
Charge capacitor voltage ($V_{C_{ivcl}}$) behavior when a 4 ms tone of 134.2 kHz is applied.

Figure 11 presents the voltage at capacitor $C_{ivcl}$. The vertical axis of this image shows the capacitor voltage in volts and the horizontal axis the time in milliseconds. $C_{ivcl}$ is used basically as energy accumulator. As expected, this capacitor with a value of 330 nF is charged to 6 V first in shallowest sensor nodes than deepest ones. The first sensor node requires 324.4 ms and the last one needs 1.278 s. As appointed in the previous paragraph, as soon as the power transmission finishes and the compensation capacitors, transmit, and receive antennas lose their energy, the sensor node still working using the accumulated energy in capacitor $C_{ivcl}$. In this experimental setup, the $C_{5vcl}$ is the first accumulator fully discharged. The reason for this is basically, because, during the $t_{c}$ period, the energy available in the compensation capacitors, the transmission, and receiving antennas is lower as the depth of the sensor node increases.

**Figure 12:**
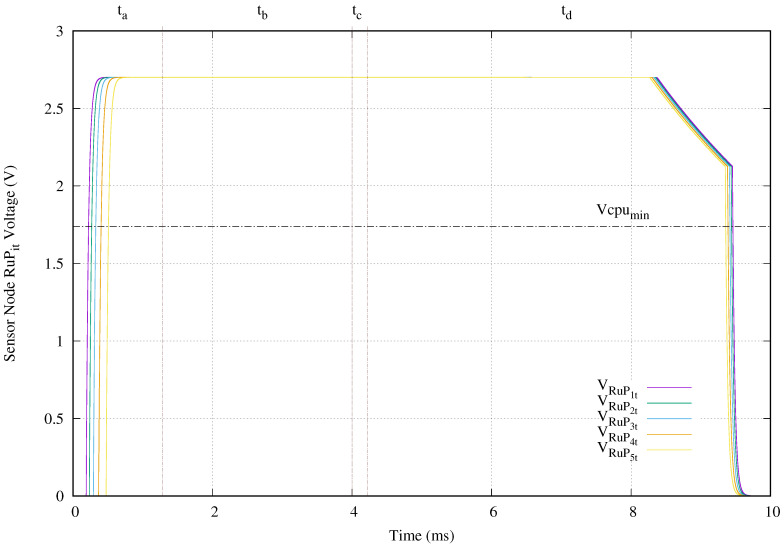
Microcontroller voltage ($V_{RuP_{it}}$) obtained when a 4 ms tone of 134.2 kHz is applied.

Figure 12 exhibits the voltage applied to the microcontroller modeled with resistance $RuP_{it}$. Its vertical axis presents the voltage in volts and its horizontal axis gives the time in microseconds. The selected microcontroller MKL17Z256 requires a supply voltage range of [1.74,2.7] V to run in ultra low power. In this sense, the upper bound is ensured with the LDO operation at 2.7 V. The lower bound of 1.74 V is determined by the availability of energy in capacitor $C_{ivcl}$. Once the $C_{ivcl}$ reaches the minimum required input voltage (2.5 V) of the LDO, it begins supplying energy to the microcontroller. The first sensor node only requires 0.22 ms and the deepest one needs 0.51 ms. Those times are lower than first period $t_{a}$. In this scenario, the microcontroller continues running until the $C_{ivcl}$ voltage drops to 2.5 V during discharge period $t_{d}$. The worst case is produced in the deepest sensor node. The microcontroller is shutdown after 9.36 ms. In this experiment setup, a 4 ms power transmission tone provides an execution time of 8.85 ms, where 54.8% of this execution time is done without external power. The best running time is 9.24 ms, which correspond to the deepest sensor node.

### 7.4. Failure Operation

The most common failure is cable breakage, as appointed in Section 3. The high conductivity of the ocean water produces a short circuit when this failure happens. This scenario can be modeled in our proposal adding on purpose a short circuit to the compensation capacitors $C_{ia},\;i\in \left[2,N\right]$.

**Figure 13:**
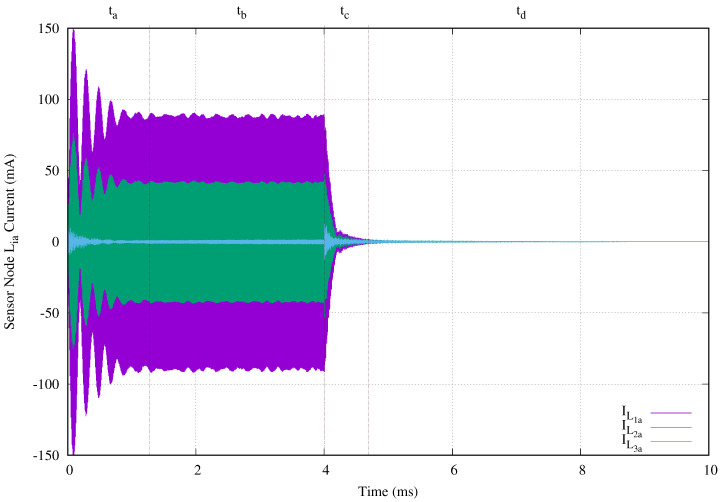
Currents in each transmission antenna of a network branch with five stages when a 4 ms tone of 134.2 kHz is applied and there is a cable breakage at $C_{4a}$.

We now suppose a cable breakage in wireless area 4 in order to evaluate the effect of this failure. We use the previous experiment setup forcing a short circuit in $C_{4a}$. Figure 13 presents the transient behavior of the currents in the transmission antennas. The vertical axis shows the current in milliamperes and the horizontal axis give the time in milliseconds. In order to clarify the image, we removed $I_{L}4a$ and $I_{L}5a$, because they are zero.

The power transmission signal is clearly visible. However, the maximum current in $L_{1a}$ is reduced from 564 mA to 150 mA when $C_{4a}$ is a short circuit. In same manner, the maximum current in the last transmission antenna located before the short circuit is substantially reduced. Its maximum value obtained from Figure 10 is 274.8 mA, and the maximum value for $I_{L_{3a}}$ is 10.8 mA as shown in Figure 13. Another effect is that energy in compensation capacitors, transmission, and receiving antennas produce a $t_{c}$ period of 0.69 ms), while normal operation only generates 0.22 ms.

**Figure 14:**
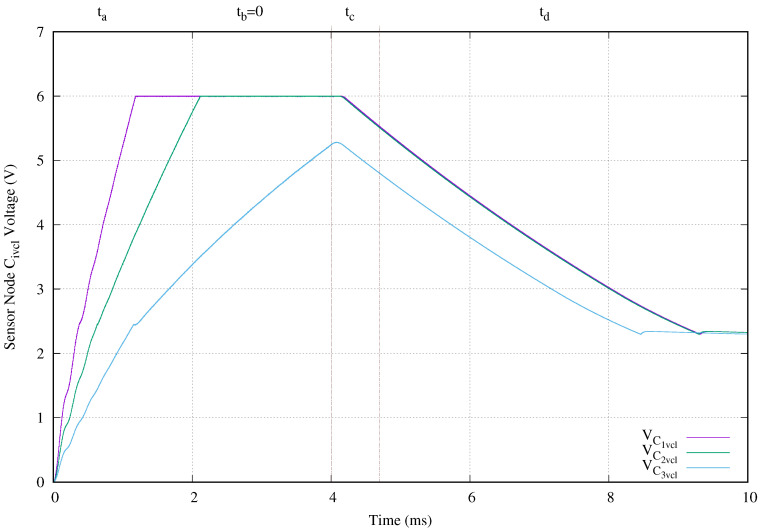
Charge capacitor voltage ($V_{C_{ivcl}}$) behavior when a 4 ms tone of 134.2 kHz is applied and there is a cable breakage at $C_{4a}$.

Figure 14 presents the voltage of the energy accumulators $C_{ivcl}$. Here, its vertical axis shows the voltage in volts and the horizontal axis the time measured in microseconds. The deepest sensor node located before the short circuit does not reach the maximum voltage (6 V) in their energy accumulator $C_{3vcl}$. The other two sensor nodes require more time in comparison with their normal operation. Those times go from 0.32 ms and 0.4 ms to 1.19 ms and 2.1 ms for the closest to ocean surface and next sensor nodes, respectively. Finally, the voltage in this accumulator reached the LDO minimum voltage level before others sensor nodes because the energy accumulator of the deepest node is not fully charged.

**Figure 15:**
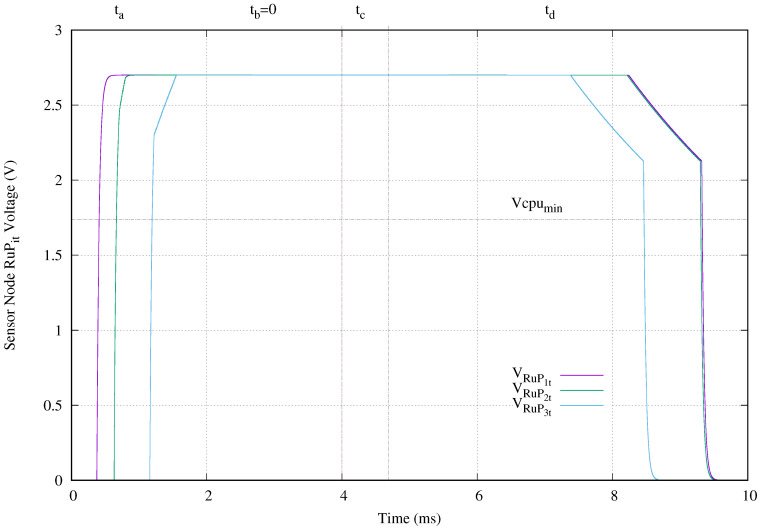
Microcontroller voltage ($V_{RuP_{it}}$) obtained when a 4 ms tone of 134.2 kHz is applied and there is a cable breakage at $C_{4a}$.

Figure 15 presents the voltage applied to the microcontroller. In spite of the slow charging of accumulator $C_{3vcl}$, the microcontroller of the sensor node located before the short circuit is powered at 1.19 ms and shut down at 8.47 ms. That is a total running time of 7.28 ms. The other sensors nodes are powered at 0.41 ms and 0.66 ms and shutdown at 9.33 ms and 9.31 ms. In other words, the closest to the ocean surface and the next sensor nodes run 8.92 ms and 8.65 ms, respectively.

In comparison with normal operation, when a cable breakage happens in the cable connection at the second deepest section, the maximum running time is only reduced from 9.24 ms to 8.92 ms for the closest to the ocean surface sensor node. This reduction is only of 3.46%. The worst case appears in the running time for the sensor node located just before the cable breakage. In this case, its run time decreases from 9.09 ms to 7.28 ms, which is 19.9% less. The second closest to the ocean surface sensor node reduces its running time a 8.3%.

In our setup, we choose the 4 ms value for power transmission time to push up to the limit the charging process of our proposed topology and show its effect. When the power transmission time is set to the minimum charging time specified in the 11784/11785 HDX standard which is 20 ms, the maximum reduction of the run time is in worst case only 4.07% less. Table 4 shows all the values for power time, shutdown time, and run time obtained from simulations and presented in Figure 12 and Figure 15.

The results from the experimental setup demonstrate the robustness of the proposed system when a short circuit happens, in comparison with traditional underwater wired sensor networks. It is well known that a cable breakage in a wired solution disables all sensor nodes that are connected to the faulty network branch. In the same scenario, the proposed mixed wireless–wired network allows to keep running the nodes that are located before the cable breakage.

When a cable breakage occurs, the cabled solution requires removing the entire network branch. Subsequently, it is repaired on land and it is finally deployed in the ocean cage. When this fatal event appears, the proposed solution only needs to remove and replace the broken section. Both tasks can be executed at same diving operation time. In addition to the cost of a single section of our proposal is much cheaper than the entire network branch of the wired solution, reducing the required diving time reduces its cost.

## 8. Conclusions

This work aims to solve the cable breakage problem of underwater sensor networks for offshore fish farm cages. We propose replacing the wired branches with a mixed wireless–wired topology. The approach is composed of modular wireless–wired sections allowing for a fast deployment and an easier maintenance in underwater diving operations. The circuital model for the proposed network branch is obtained, and the analytical model for wireless power transfer (WPT) design and the efficiency optimization are presented. Therefore, the circuit conditions for a perfect impedance adaptation of the entire network are obtained; the formulas for the compensation capacitor values of the WPT network are demonstrated. In addition, the influence of parasitic resistances on the performance of the complete network is analyzed in detail.

Our experimental network branch includes: two circular inductors of 452 μH as antennas operating at 134.2 kHz, using the ISO 11784/11785 HDX standard, and a sensor node antenna using a SMD ferrite core inductor of 2.66 mH. The mutual inductances for the proposed setup are $M_{iab}$ = 316.16 μH for the transmission and receiving antennas, when they are placed within a distance of 200 μm, which provides a coupling factor of $K_{iab}$ = 0.6996. The mutual inductance between the transmission and sensor node antennas is $M_{iat}$ = 73.5 μH and the equivalent coupling factor is $K_{i}$ = 0.06632.

Because of the low parasitic resistance of the sensor node antenna, 26 Ω, its influence is neglected. Using this low parasitic resistance values in sensor nodes of our network branches, we demonstrated that the load impedance is greater than the sensor node antenna parasitic resistance by more than three orders of magnitude. This scenario defines an upper and lower bound of the transmitted current for the last load. We obtained for the proposed setup that the maximum current is 48.71 mA. In the worst case, when all parasitic resistances from all of the transmission antennas are taken into consideration, the maximum transmitted current to the load is 1.864 mA.

The proposed topology is applied to an experimental setup, including up to five sensor nodes, so that from a sensor to the following one there exists a six meters of cable connecting them. Therefore, the total length of mixed wireless–wired network is 30 m in the experiment setup. In this configuration the power is transferred efficiently to the last sensor node, such that a battery of 3 mAh is fully charged in less than 97 minutes in the worst case scenario.

After demonstrating the proposal compliance with the ISO 11784/11785 HDX standard in its normal operation, we evaluated the approach behavior when a cable breakage happens. We forced a cable breakage and the proposed topology keeps running the sensor nodes located before the short circuit in order to simulate this fatal scenario. The sensor nodes run time is reduced only a maximum of 4.07% using the ISO standard values when the breakage is located at the second deepest section.

## Figures and Tables

**Table 1:** Experiment Setup for Parasitic Resistance Evaluation in the Last Sensor Node.

Parameter	Value
N	5
Total Depth	30 m
*V*	5 Vpp
*f*	134.2 kHz
$L_{ia}$ and $L_{ib}$	452 μH
$M_{iab}$	316.16 μH
$M_{5at}$ and $M_{5bt}$	73.5 μH
$C_{ja}$ and $C_{ib}$	3.1117 nF
$C_{5a}$	3.0978 nF
$L_{jt}$, $R_{jt}$, $C_{jt}$ and $Z_{jt}$	−${}^{*1}$
$L_{i5t}$	2.66 mH
$C_{5t}$	526.489 pF
$Z_{5t}$	15k Ω
$R_{5t}$	26 Ω
Loop Resistance 23 AWG	0.744 Ω
Loop Resistance 8 AWG	0.024 Ω
$R_{ia}$ and $R_{ib}$ (38 AWG)	9.3 Ω
$R_{ia}$ and $R_{ib}$ (23 AWG)	248.16 mΩ

i∈[1,5] and j∈[1,4]; ${}^{*1}$ Only sensor node 5 is present.

**Table 2:** Experiment Setup for Frequency Response Evaluation of a network branch with five stages.

Parameter	Value
N	5
Total Depth	30 m
*V*	5 Vpp
$L_{ia}$ and $L_{ib}$	452 μH
$M_{iab}$	316.16 μH
$M_{iat}$ and $M_{jbt}$	73.5 μH
$C_{ja}$ and $C_{ib}$	3.1117 nF
$C_{5a}$	3.0978 nF
$L_{it}$	2.66 mH
$C_{it}$	526.489 pF
$Z_{it}$	15k Ω
$R_{it}$	26 Ω
$R_{ia}$ and $R_{ib}$	620.16 mΩ${}^{*1}$

i∈[1,5] and j∈[1,4]; ${}^{*1}$ using 23 AWG standard.

**Table 3:** Sensor node components values.

Parameter	Value
$L_{it}$	2.66 mH
$R_{it}$	26 Ω
$C_{it}$	526.489 pF
$D_{it}$	$V_{f}$ = 715 mV
$C_{ivcl}$	330 nF
$Dz_{it}$	$V_{z}$ = 6 V
$IC_{iLDO}$	TPS71727
$C_{iNR}$ and $C_{iRuP}$	10 nF
$RuP_{it}$	15k Ω

i∈[1,5].

**Table 4:** Microcontroller powering, shutdown, and run times under normal and failure operations.

T	Sensor	Normal			Failure			Reduction
		$t_{\mathrm{on}}$	$t_{\mathrm{sd}}$	$t_{\mathrm{rt}}$	$t_{\mathrm{on}}$	$t_{\mathrm{sd}}$	$t_{\mathrm{rt}}$	
4	1	0.22	9.46	9.24	0.41	9.33	8.92	5.7
	2	0.27	9.44	9.17	0.66	9.31	8.65	8.3
	3	0.33	9.42	9.09	1.19	8.47	7.28	19.9
	4	0.40	9.39	8.99	-	-	-	-
	5	0.51	9.36	8.85	-	-	-	-
20	1	0.22	25.46	25.24	0.41	25.32	24.91	1.3
	2	0.27	25.44	25.17	0.66	25.30	24.64	2.1
	3	0.33	25.42	25.09	1.19	25.26	24.07	4.07
	4	0.40	25.39	24.99	-	-	-	-
	5	0.51	25.35	24.84	-	-	-	-

T power transmission, $t_{on}$ powered, $t_{sd}$ shutdown and $t_{rt}$ run times.
